# Viral co‐infection with human respiratory syncytial virus in suspected acute and severe respiratory tract infections during COVID‐19 pandemic in Yaoundé, Cameroon, 2020–2021

**DOI:** 10.1111/irv.13131

**Published:** 2023-03-29

**Authors:** Moïse Henri Moumbeket Yifomnjou, Gwladys Chavely Monamele, Mohamadou Njankouo‐Ripa, Abdou Fatawou Modiyinji, Paul Alain Ngoupo, Onana Boyomo, Richard Njouom

**Affiliations:** ^1^ Centre Pasteur of Cameroon Yaounde Cameroon; ^2^ Laboratory of Microbiology University of Yaounde I Yaounde Cameroon

**Keywords:** acute respiratory infections, Cameroon, co‐infections, influenza, RSV, SARS‐CoV‐2

## Abstract

**Background:**

Acute lower respiratory tract infections (ALRIs) are one one of the leading causes of morbidity and mortality among people of all ages worldwide, particularly in low‐ and middle‐income countries (LMICs). The purpose of this study was to determine epidemiological characteristics of respiratory viruses in acute respiratory infection (ARI) patients during the COVID‐19 pandemic in Yaoundé, Cameroon.

**Methods:**

Patients were monitored for respiratory symptoms as part of the surveillance of severe acute respiratory syndrome coronavirus 2 (SARS‐CoV‐2) and other respiratory viral infections. Patients of all ages with respiratory symptoms less than 5 days were considered. Sociodemographic and clinical data as well as nasopharyngeal samples was collected from patients. Nasopharyngeal samples were tested for SARS‐CoV‐2, influenza, and respiratory syncytial virus (RSV) using real‐time reverse‐transcription polymerase chain reaction methods. Virus distribution and demographic data were analyzed with R version 2.15.1.

**Results:**

From July 2020 to October 2021, 1120 patients were included. The overall viral detection rate was 32.5%, including 9.5% for RSV, 12.6% for influenza virus and 12.8% for SARS‐CoV‐2. Co‐infections were detected in 6.9% of positive cases. While RSV and influenza virus showed seasonal trends, SARS‐CoV‐2 was detected throughout the study period.

**Conclusion:**

We found that during COVID‐19 pandemic, respiratory viruses play an important role in etiology of influenza‐like illness in Cameroon, and this observation was true for patients of all ages.

## 1 INTRODUCTION

Globally, acute lower respiratory tract infection (ALRI) remains one of the leading causes of morbidity, hospitalization, and mortality in people of all ages.^1^, ^2^, ^3^, ^4^ Data from a systematic review shows that in 2016, approximately 336 million episodes of ALRI in people of all ages resulted in over 65 million hospitalizations with an overall mortality of 2.3 million deaths.^5^ Human respiratory syncytial virus (HRSV) is one of the most common viral pathogens identified in ALRI, particularly in children under 5 years and adults over 65 years.^6^, ^7^ RSV infection can cause mild symptoms, such as fever, cough, wheezing, and also severe symptoms, including bronchiolitis and pneumonia.^8^ Episodes of RSV‐ALRI have been estimated to be responsible for 76,612 (95% CI, 55121–103,503) deaths, making RSV the second leading etiology of deaths due to lower respiratory infection worldwide.^5^ Thus, RSV infection, in addition to having a real impact on health, also has a significant socioeconomic impact on resources (visits to primary care and specialized centers, emergency rooms, hospitalizations, admissions to intensive care units, diagnostic tests, and treatments), costs (direct and indirect costs), quality of life, and work absenteeism, which is higher among parents of children under 3 years old.^9^, ^10^, ^11^ RSV is a single‐stranded, enveloped, negative‐sense, helical‐capsid ribonucleic acid (RNA) virus belonging to the Mononegavirales order, Pneumoviridae family, and *Orthopneumovirus* genus.^12^ Initial RSV infection may coincide with another viral infection, causing what is called co‐infection.^13^ Thus, many other viruses such as influenza and SARS‐CoV‐2 have been involved in co‐infections with RSV, often increasing the risk of developing acute respiratory infection (ARI).^14^

In 2007, “Centre Pasteur Cameroon (CPC)” was designated National Influenza Centre (NIC) by the Ministry of Public Health, Cameroon. Since then, surveillance of influenza and other respiratory pathogens including RSV has been performed in routine. This has led to better understanding of epidemiology of major respiratory pathogens in the country through numerous studies conducted and published.^13^, ^15^, ^16^, ^17^, ^18^ For example, a study to describe 10 years of influenza surveillance data in Cameroon between January 2009 and December 2018 reported an overall positivity rate of 24% with year‐round circulation.^17^ On the other hand, for RSV between 2011 and 2013, a study reported a positivity rate of 13.2% with seasonal circulation.^13^ In 2020, with the advent of the COVID‐19 pandemic, CPC was the first laboratory to start detection of SARS‐CoV‐2 cases owing to experience gained during the implementation of influenza surveillance. Due to the year‐round circulation of influenza and RSV in Cameroon, the probability of co‐infections between SARS‐CoV‐2 and other respiratory pathogens was likely in Cameroon. Also, with mitigation measures that increased during the COVID‐19 pandemic, it is important to study the impact of public health measures in the circulation of other respiratory pathogen in general and particularly in influenza, which is a vaccine‐preventable virus, and on RSV.

## 2 MATERIALS AND METHODS

### 2.1 Study design

Administratively, Cameroon is organized into 10 regions. Among these regions is the Central region with Yaoundé as its capital. Despite climatic changes observed in recent years, this region is often characterized by an equatorial Guinean type climate with four seasons: two rainy seasons and two dry seasons.

We conducted a descriptive study from March 2020 to October 2021. This study was carried out on one hand in 05 influenza sentinel sites in Yaoundé city established for influenza surveillance since 2007 and in the other hand, at CPC, which was established for surveillance of SARS‐CoV‐2 in 2020. These sentinel sites composed of public (three) and private (two) primary care centers as well as CPC are all located in Yaoundé city, Centre region of Cameroon. Patient's recruitment happened differently between sites and CPC. As far as sentinel sites are concerned, qualified medical personnel identified all influenza‐like illness (ILI) and severe acute respiratory infection (SARI) cases presenting daily in hospitals. ILI or SARI case was determined using a WHO case definition. An ILI case is an ARI with the following symptoms: fever measured ≥38°C, cough, with onset within the past 10 days, whereas a SARI case is an ARI with the following symptoms: fever or fever measured ≥38°C, cough, with onset within the past 10 days and requiring hospitalization.^19^ Verbal informed consent was obtained for all identified cases aged ≥ 18 years. For patients under age of 18, informed consent by proxy was obtained from parents or legal guardians. Regarding specimens that were collected as part of SARS‐CoV‐2 surveillance and sent at the CPC, inclusion criteria was based on the national guidelines for case definition of COVID‐19 in Cameroon adopted by all collection sites.

### 2.2 Ethical considerations

This study was reviewed and approved by the Centre Regional Human Health Research Ethics Committee and Cameroon Ministry of Public Health.

### 2.3 Study and laboratory procedures

Sociodemographic and clinical information was obtained from participants using a standardized questionnaire.

For samples originating from influenza sentinel sites, nasopharyngeal and/or oropharyngeal swab specimens were collected from all included patients and transported to CPC in cooler containing ice packs. At CPC, total RNAs were extracted using the QiAMp Viral RNA Extraction Kit as previously described.^13^ Purified RNA was stored at −80°C. Detection of influenza, RSV, and SARS‐CoV‐2 was performed using real‐time RT‐PCR methods. Identification of types and subtypes of seasonal influenza viruses was performed using methods developed by US Center for Disease Control (CDC) and Prevention. Then, SARS‐CoV‐2 virus detection was performed on the same samples using one of the following four real‐time RT‐PCR protocols: Sansure Biotech Inc. (Hunan, China; Doc. #: 2019‐nCoV IFU), Da an Gene Co., Ltd. of Sun Yat‐sen University (China; EUL 0493‐141‐00), Abbott Molecular Inc. (USA, Abbott RealTime SARS‐CoV‐2 assay, EUL‐0503‐027‐00), Applied Biosystems by ThermoFisher Scientific (USA, TaqPath™ COVID‐19 CE‐IVD RT‐PCR Kit, A48067). Meanwhile, detection of RSV was carried out according to methods developed by US CDC and Prevention. PCR amplifications were performed on Applied Biosystems™ 7500 Fast Real‐Time PCR Instrument, Applied Biosystems™ QuantStudio™ 7 Flex Real‐Time PCR instrument 96 well‐block, Abbott m2000rt, or LightCycler™ 96. A sample was considered positive for one of the viruses if the cycle threshold was below the threshold value of 36.

### 2.4 Data management and statistical analysis

Information from the questionnaire and laboratory results were saved in a Microsoft® Excel database (Microsoft, Washington, DC, USA). We analyzed demographic and clinical characteristics of study subjects, as well as frequency and seasonal patterns of the three respiratory viruses. Student's *t*‐test, Mann–Whitney U‐test, and Kruskal–Wallis rank test were used for description of continuous variables, and Fisher's exact test was used for description of categorical variables. We analyzed clinical characteristics and status of participants with respect to the presence or absence of respiratory infection using a stepwise logistic regression model. *p*‐values <0.05 were considered statistically significant for all parameters. Uncertainty was expressed as 95% confidence intervals (CIs). Statistical analysis was carried out with R program version 2•15•1.

## 3 RESULTS

### 3.1 Demographic and clinical characteristics of patients with ILI

During the period from July 2020 to October 2021, 1120 patients with ILI or SARI were included (Table 1). Among these, 40.4% (453/1120) of patients included came from influenza surveillance, whereas 59.6% (667/1120) were from SARS‐CoV‐2 surveillance. Age of patients ranged from 1 month to 88 years with median age of 32 years (IQR: 2–48 years). Most patients (62%) were older than 5 years. In addition, 295 (26.4%) were younger than 5 years and up to 131 (11.7%) patients had no specified age. There were 510 males (45.5%), 491 females (43.8%), and 119 (10.6%) unspecified gender.

**TABLE 1:** Demographic and clinical characteristics of outpatients and hospitalized patients with influenza‐like illness (ILI) and 1 or >1 respiratory virus detected, Yaoundé, Cameroon, 2020–2021.

		Respiratory virus(es) detected		
Characteristics	ILI detected (*n* = 1120)	1 (*n* = 339)	>1 (*n* = 25)	Total (*n* = 364)
Sex				
Men	510 (45.5)	150 (44.2)	10 (40.0)	160 (43.9)
Women	491 (43.8)	158 (46.6)	13 (52.0)	171 (47.0)
Missing	119 (10.6)	31 (9.1)	2 (8.0)	33 (9.1)
Age				
<1 year	114 (10.2)	43 (12.7)	3 (12.0)	46 (12.6)
1–5 years	181 (16.2)	69 (20.4)	7 (28.0)	76 (20.9)
5–15 years	52 (4.6)	22 (6.5)	2 (8.0)	24 (6.6)
15–30 years	118 (10.5)	27 (8.0)	0 (0)	27 (7.4)
30–65 years	441 (39.4)	118 (34.8)	9 (36.0)	127 (34.9)
>65 years	83 (7.4)	29 (8.6)	2 (8.7)	31 (8.5)
Missing	131 (11.7)	31 (9.1)	2 (8.0)	33 (9.1)
Symptom				
Cough	578 (51.6)	211 (62.2)	21 (84)	232 (63.7)
Rhinorrhea	419 (37.4)	161 (47.5)	14 (56)	175 (48.1)
Sore throat	218 (19.5)	75 (22.1)	6 (24)	81 (22.3)
Tiredness	204 (18.2)	66 (19.5)	1 (4)	67 (18.4)
Headache	168 (15)	75 (22.1)	4 (16)	79 (21.7)
Shortness of breath	140 (12.5)	47 (13.8)	2 (8)	49 (13.5)
Myalgia	110 (9.8)	44 (12.9)	4 (16)	48 (13.2)
Conjunctivitis	91(8.1)	28 (8.3)	1 (4)	29 (7.9)
Arthralgia	81 (7.2)	24 (7.1)	2 (8)	26 (7.1)
Wheezing	62 (5.5)	27 (7.9)	2 (8)	29 (7.9)
Vomiting	54 (4.8)	19 (5.6)	0 (0)	19 (5.2)
Diarrhea	51 (4.6)	18 (5.3)	0 (0)	18 (4.9)
Ear pain	25 (2.2)	8 (2.4)	0 (0)	8 (2.2)
Skin rash	22 (2)	2 (0.6)	1 (4)	3 (0.8)
Source				
Influenza surveillance	453 (40.4)	168 (49.6)	14 (56)	182 (50)
SARS‐CoV‐2 surveillance	667 (59.6)	171 (50.4)	11 (44)	182 (50)

*Note*: Data are represented as numbers and percentages are in parentheses. Inclusion criterion was fever; therefore, all patients included were febrile.

**FIGURE 1:**
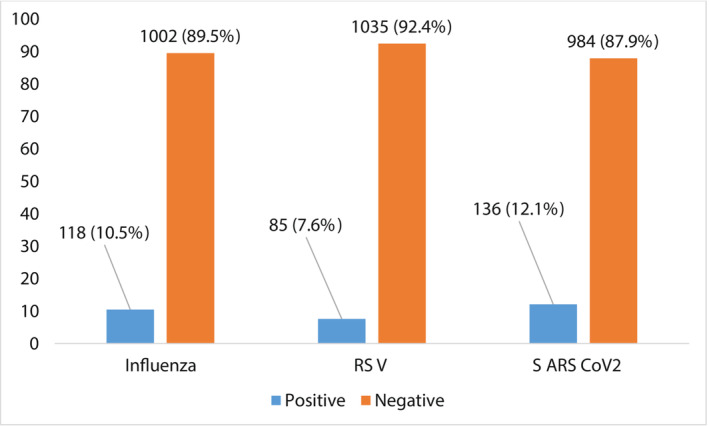
Number of each respiratory virus/group of viruses detected as unique infections.

**FIGURE 2:**
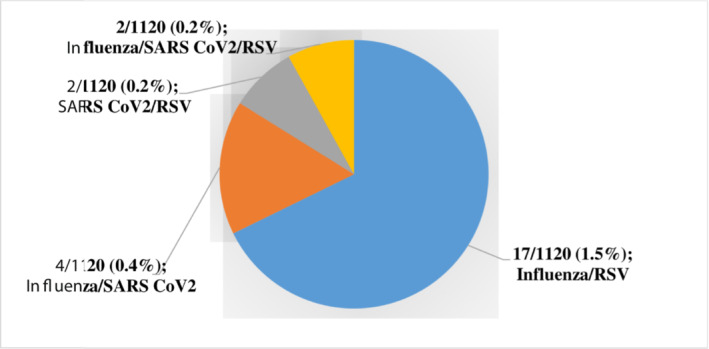
Number of each respiratory virus/group of viruses detected as mixed infections.

All patients presented with fever, as that was inclusion criterion. Cough was present in most cases (51.9%, 578/1120) (Table 1). Of the 1120 samples tested, 364 (32.5%) were positive for at least one viral pathogen (Figure 1). Among the 364 positive samples, a single infection was detected in 339 (93.1%) samples, whereas co‐infections were found in 25 (6.9%) samples tested including 23 double infections and two triple infections (Figure 2). Single infections and co‐infections occurred in all age groups except 15–29 years, which had no cases of co‐infection. Furthermore, highest percentage of viral infections was observed in patients aged 30–65 years (34.9%, 127/364). Of the 127 infections in these patients aged 30–65 years, 118 (92.9%) were single infections and nine (7.1%) were co‐infections.

### 3.2 Virus detection

SARS‐CoV‐2 and influenza were the most frequently identified viruses in ILI patients, with frequencies of 144/1120 (12.8%) and 141/1120 (12.6%), respectively, followed by RSV with a frequency of 106/1120 (9.5%). Of the 141 influenza viruses detected, 127 (90.1%) were influenza A viruses (80 H1N1 [A (H1N1) pdm09], 47 A [H3N2]), and 14 (9.9%) were influenza B viruses (Table 2).

**TABLE 2:** Detection rate and age‐specific (year) distribution of each detected virus or virus group and co‐infection rate.

Virus	<1 (*n* = 114)	[1–5] (*n* = 181)	[5–15] (*n* = 52)	[15–30] (*n* = 118)	[30–65] (*n* = 441)	>65 (*n* = 83)	Missing (*n* = 131)	Total (*n* = 1120)
Influenza								
Any	16 (14)	50 (27.6)	20 (38.4)	11 (9.3)	30 (6.8)	2 (2.4)	12 (9.1)	141 (12.6)
Type A	15 (13.2)	48 (26.5)	15 (28.8)	10 (8.5)	27 (6.1)	2 (2.4)	10 (7.6)	127 (11.3)
Type B	1 (0.8)	2 (1.1)	5 (9.6)	1 (0.8)	3 (0.7)	0	2 (1.5)	14 (1.3)
VRS	32 (28.1)	33 (18.2)	4 (7.7)	3 (2.5)	15 (3.4)	6 (7.2)	13 (9.9)	106 (9.5)
SARS‐CoV‐2	1 (0.8)	0	2 (3.8)	13 (11)	92 (20.8)	26 (31.3)	10 (7.6)	144 (12.8)
Monoinfection	43 (37.7)	69 (38.1)	22 (42.3)	27 (22.8)	118 (26.7)	29 (34.9)	31 (23.7)	339 (30.3)
Co‐infection	3 (2.6)	7 (3.8)	2 (3.8)	0	9 (2)	2 (2.4)	2 (1.5)	25 (2.2)
Total	46 (40.3)	76 (41.9)	24 (46.1)	27 (22.8)	127 (28.7)	31 (37.3)	33 (25.2)	364 (32.5)

RSV was the most frequently detected virus in children younger than 1 year of age (28.1%, 32/114), followed by influenza A virus (14%, 16/114) and SARS‐CoV‐2 (0.8%, 1/114). On the other hand, in children aged 1–4 years, influenza A virus was the most frequently detected virus (27.6%, 50/181), followed by RSV (18.2%, 33/181), and there were no cases of SARS‐CoV‐2 detection. However, in age groups ≥5 years, SARS‐CoV‐2 (17.3%, 143/825) was the most frequently detected virus with higher detection rates (11.2%, 92/825) in the age group 30–65, followed by influenza A virus (9.1%, 75/825) and then RSV (4.9%, 41/825).

In a downward Wald binary logistic regression, RSV positive specimens were negatively associated only with rhinorrhea (*p* = 0.017, odds ratio [OR], 12.48; 95% CI, 1.55–99, 98), whereas SARS‐CoV‐2 positive specimens were only negatively associated with myalgia (*p* = 0.042, odds ratio [OR], 10.95; 95% CI, 1.08–110.34). No other clinical symptoms were associated with positive patients, nor with any other specific virus detected or co‐infection.

Data are no. (%) of patients.

### 3.3 Temporal distribution of respiratory viruses

**FIGURE 3:**
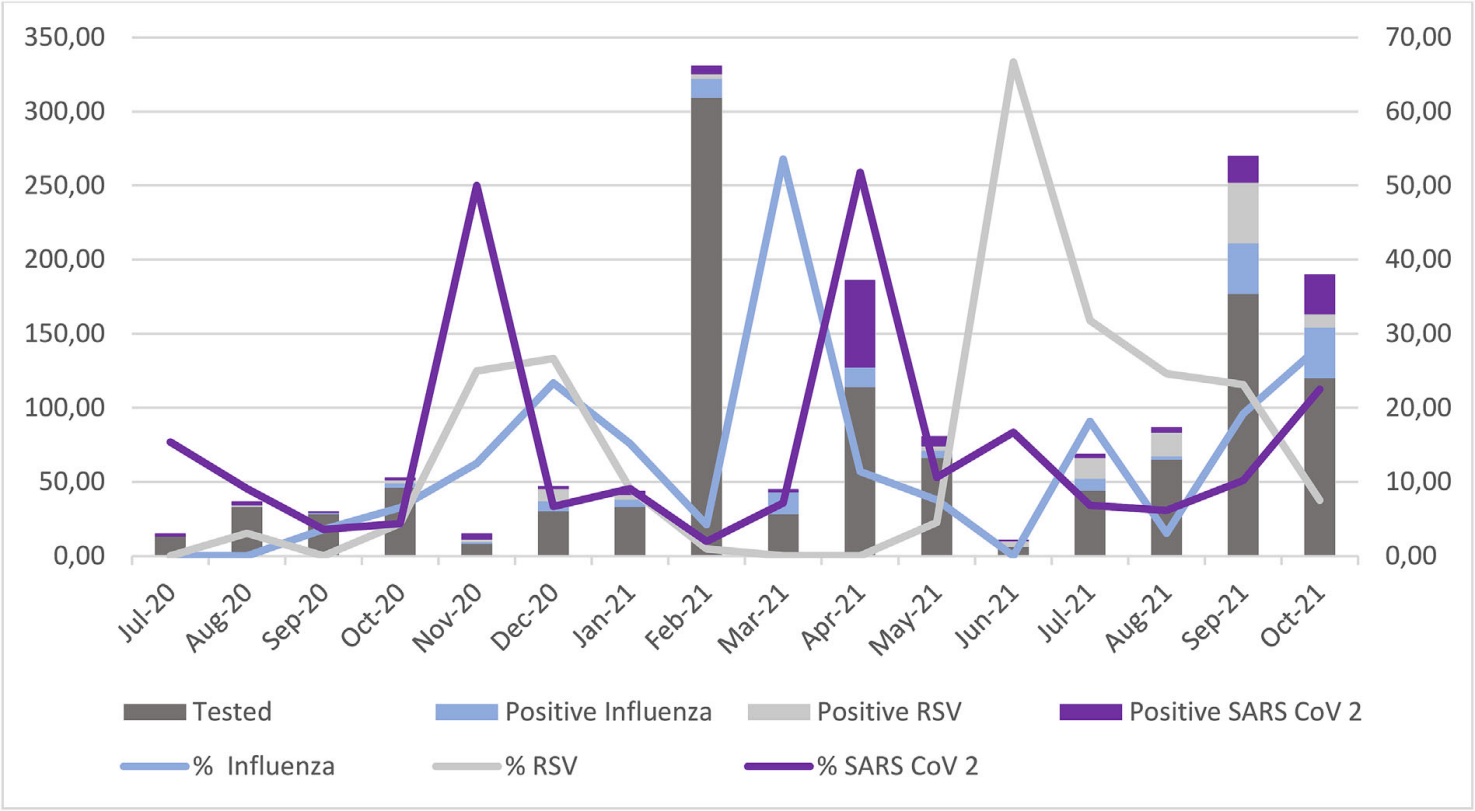
Temporal distribution of respiratory viruses or group of viruses detected in individuals with influenza‐like illness (ILI) from July 2020 to October 2021. The *y*‐axis and primary bars describe the number of cases, whereas *y*‐axis and secondary lines describe the monthly detection rate.

All three viruses circulated were observed throughout the study period. Figure 3 shows temporal distribution of virus detected. RSV showed higher peaks observed in December 2020 and June 2021. SARS‐CoV‐2 was detected throughout the study period with a peak of activity observed in November 2020 and April 2021. Peak in influenza virus was mainly observed in October to December 2020, and in March and July 2021.

## 4 DISCUSSION

This study is the first to describe the frequency and distribution of Respiratory Syncytial Virus and influenza virus associated with ILI patients in Cameroon during the COVID‐19 pandemic. The data presented here are unique to our knowledge. Respiratory viruses were detected in 32.5% of samples; SARS‐CoV‐2 and influenza virus were the most frequently detected viruses. Almost moderately high frequency of virus detection in patients with ILI during the pandemic in our study is consistent with results of other etiological studies conducted in similar settings,^20^, ^21^, ^22^, ^23^, ^24^ Nevertheless, this proportion seems to vary widely from one country to another. Si et al. recorded 10.3% in China^25^ and Hirotsu et al. recorded 17% in Japan.^26^ Higher percentages of 52.3% were reported in Italy,^27^ and 70% in China^28^ were respectively reported by Calderado et al. and Li et al. Possible reasons for these variable results between studies include true differences in epidemiology, patients age, climate, differences in inclusion criteria for study population, panel of viral agents tested, diagnosis methods used, study duration. It also shows that this frequency of 32.5% in this study is lower than those obtained in previous studies conducted in Cameroon during the pre‐pandemic period, which reported detection frequencies of 65.1% and 65.4%, respectively.^13^, ^18^ Apart from the above reasons, non‐pharmaceutical interventions such as social distancing, face masks, and increased hand hygiene used to limit the spread of COVID‐19 could also explain the decrease in respiratory viral infections.

As in many other studies, SARS‐CoV‐2 was the most common viral pathogen detected in ILI patients, representing 12.8% viruses detected. This value is lower than that (21%, 21.2%, 22.9%, 28.3%, and 34.6%) reported in most other similar studies.^29^, ^30^, ^31^, ^32^, ^33^ It is nevertheless similar to what was noted in California, Cairo, and Illinois^34^, ^35^, ^36^ by Kim et al. (9.5%), Fahim et al. (12%), and Hazra et al. (18.1%), respectively; and higher than that reported in Australia and southwest China.^21^, ^25^ Additionally, influenza and RSV were identified from patient samples and collectively contributed to 18.1% of all ILI cases in our study. RSV detection rate in our study (9.5%) was lower than rates reported by other investigators. For example, in studies from Italy^27^ and China,^37^ 23.7% and 31.8% of samples from patients with ILI were positive for RSV, respectively. However, studies by researchers such as Si et al.,^25^ Hirotsu et al.,^26^ Calcagno et al.,^32^ Chong et al.,^33^ Kim et al.,^34^ and Hazra et al.^36^ reported similar but lower values than our study for detection rates ranging from 0.5% to 3.2%. Small proportion of RSV positive samples in our study may be due to the fact that most patients (62%) were older than 5 years, as RSV has been shown to be a common cause of lower respiratory tract infection in children under 5 years.^6^, ^38^ Thus, RSV generally spreads among children, and attendance at day care centers has markedly decreased during pandemic period in Cameroon. This result suggests that school closures, curfews for children, and smaller day care groups among younger children can reduce burden of disease and hospitalizations caused by RSV.

During the first year of the pandemic, influenza surveillance system was greatly affected as the same actors were requisitioned to perform SARS‐CoV‐2 surveillance, and thus, there was a marked decrease in activities and consequently decreasing influenza positivity rates. Influenza positivity rate of 12.6% was observed during this study, which is lower in 2007; CPC was designated NIC by the Ministry of Public Health, Cameroon, and also lower than a study that aimed to describe 10 years of influenza activity in Cameroon.^17^, ^18^ Olsen et al.^39^ also saw a decrease in influenza activity during COVID‐19 pandemic in United States, Australia, Chile, and South Africa as well Yum et al. in South Korea^20^ and Agca et al. in Turkey.^22^ These low proportion detection rates were similar to that reported in other studies (0.5%–10.5%).^25^, ^27^, ^32^ However, contrary to our results, some similar studies have shown that influenza virus remains one of the most common viral pathogens detected during the pandemic.^37^ Apart from decrease in tests in our laboratory due to fact that during pandemic health professionals preferentially referred patients for a screening test for SARS‐CoV‐2. Another reason that could explain decrease in influenza activity can be attributed to viral interference, which is usually caused by another respiratory virus which could outcompete influenza in respiratory tract.^40^

Identifying multiple respiratory virus co‐infections can provide insight into different clinical symptoms, long‐term health effects, and appropriate prevention methods. In case of viral respiratory tract infections leading to pneumonia, mixed infection can lead to severe illness in patients with compromised immune systems.^41^ Moreover, due to similarity in clinical symptoms shared by other respiratory viruses, it has become quite difficult to accurately distinguish SARS‐CoV‐2 infection from other respiratory viral infections.^42^ In this study, respiratory viral co‐infections were identified in 6.9% of positive cases with ILI. During the pre‐pandemic period, studies published in Cameroon particularly and Africa as a whole reported wide range of prevalence of viral‐coinfections.^13^, ^18^, ^43^, ^44^ However, results from this study is similar to that reported in other countries, including Malaysia (2.5%),^33^ France (6%),^29^ and United States (1.5%)^45^ but lower than that reported by other studies (10% and 20%).^34^, ^46^ This could be due different study populations or potential spatio‐temporal variations in viral epidemiology.

Pandemic development is dynamic and dominated by immunological naivety world population to SARS‐CoV‐2. This study found that SARS‐CoV‐2 infections in Cameroon did not have an overall distribution similar to influenza and RSV. Although SARS‐CoV‐2 is the most frequently detected virus in this study, higher rates were observed in March 2020 and April 2021. In general, these three respiratory viruses exhibited different temporal distribution during study period. The observed distribution differed from that reported in published Cameroonian studies describing pathogens other than SARS‐CoV‐2.^13^, ^16^, ^18^ Although there may be year‐to‐year heterogeneity in viral prevalence, this study suggests significant heterogeneity across the country and uncovers the importance of large‐scale surveillance that extends beyond the Centre region. Interventions that take into account local epidemiology and infection patterns are needed to address both seasonal respiratory infections and pandemic spread.

This study is not without limitations. First, our study included people that were tested at CPC laboratory and therefore is not representative of all people tested in Yaoundé. Secondly, we tested only three RNA respiratory viruses, no DNA respiratory viruses, and no respiratory bacteria or fungi. This would have limited full spectrum detection of present seasonal respiratory viruses and underestimated viral and/or bacterial co‐infection in patients with ILI. Third, SARS‐CoV‐2 virus testing methods have changed over time to meet pandemic needs. Differences in sensitivity and specificity between tests may exist, which may have caused some missed detections of SARS‐CoV‐2 and consequently, fewer co‐infection detections. Finally, we did not have access to all patient records and therefore relied on clinical information provided on the form, which may have prevented full exploration of disease severity.

Despite these limitations, this study is first to describe etiology of RNA viral pathogens involved in upper respiratory tract infections during COVID‐19 pandemic in Cameroon. These viruses can also induce more serious diseases, especially in patients of all ages, which must be studied.

This study should be applied in various settings and over many years to determine seasonal variation of ALRIs and individual viruses with the aim of providing a better understanding of differences in seasonality, epidemiology, and spectrum of disease caused by respiratory viruses in Cameroon. However, whether concurrent viral infection in patients with SARS‐CoV‐2 can potentially lead to viral interference or impact disease outcome awaits further research.

## AUTHOR CONTRIBUTIONS

**Moïse Henri Moumbeket Yifomnjou:** Formal analysis; methodology; writing–original draft; writing–review and editing. **Gwladys Chavely Monamele:** Investigation; writing–review and editing. **Mohamadou Njankouo‐Ripa:** Investigation; writing–review and editing. **Abdou Fatawou Modiyinji:** Investigation; writing–review and editing. **Paul Alain Ngoupo:** Investigation; writing–review and editing. **Boyomo Onana:** Conceptualization; methodology; supervision; writing–review and editing. **Richard Njouom:** Conceptualization; funding acquisition; methodology; supervision; validation; writing–review and editing.

## CONFLICT OF INTEREST STATEMENT

The authors declare that they have no competing interests.

### PEER REVIEW

The peer review history for this article is available at https://www.webofscience.com/api/gateway/wos/peer-review/10.1111/irv.13131.

## Data Availability

The data that support the findings of this study are available from the corresponding author upon request.
